# C-954-16. Utilization of Speech Pathology Services in the Burn ICU: Outcomes, Benefits, and Order Set Impact

**DOI:** 10.1093/jbcr/irag033.148

**Published:** 2026-04-06

**Authors:** Hadley A Regal, Kristina Frazier, Christopher R LaChapelle, Callie M Thompson

**Affiliations:** University of Utah Health Burn Center, Salt Lake City, UT; University of Utah Health, Salt Lake City, UT; University of Utah Health, Salt Lake City, UT; University of Utah Health, Salt Lake City, UT

## Abstract

**Introduction:**

Speech-Language Pathology (SLP) services are a vital component of interdisciplinary care in the Burn Intensive Care Unit (ICU), addressing complex impairments in communication, swallowing, and cognition. Burn injuries often result in orofacial trauma and complications that require early rehabilitative intervention. In 2021, a standardized speech therapy admission order set was implemented to improve timely access to SLP services.

**Methods:**

A retrospective review was conducted of burn ICU patients from January 1, 2017, to August 31, 2025 (n = 3187 total admits). Two cohorts were analyzed: pre-implementation (2017–2020, n = 1498) and post-implementation (2021–2025, n = 1689). In the pre-implementation group, 321 patients had SLP orders, with an overall SLP evaluation rate of 21.4%. In the post-implementation group, 1372 patients had SLP orders, increasing the evaluation rate to 81.2%. Interventions reviewed included swallowing assessments, voice therapy, cognitive-communication rehabilitation, nonverbal communication strategies, orofacial contracture management, and instrumental swallow evaluations using Modified Barium Swallow Studies (MBSS) and Fiberoptic Endoscopic Evaluation of Swallowing (FEES).

**Results:**

Following the implementation of the order set, SLP service utilization significantly increased. The post-implementation cohort demonstrated earlier access to SLP evaluation, faster return to oral intake, shorter time to discharge, and more efficient completion of SLP services (see Fig. 1). Additionally, there was a reduction in aspiration pneumonia and ICU length of stay. These improvements are associated with earlier intervention and standardized access.

**Conclusions:**

Implementation of an admission speech therapy order set significantly increased the utilization of SLP services and improved clinical outcomes in the Burn ICU. Early and consistent SLP involvement was associated with reduced complications and enhanced recovery timelines. The findings support the adoption of standardized referral protocols to ensure timely and equitable SLP access in burn care. Future research should examine long-term cost-effectiveness and the broader application of this approach across burn centers.

**Applicability of Research to Practice:**

Standardized SLP order sets improve access, reduce complications, and shorten ICU stays in burn care. Routine early referral can enhance recovery, support interdisciplinary care, and be replicated across other burn centers to promote consistent outcomes.

**Funding for the study:**

N/A.

